# Long-term outcome (5-10 years) after non absorbable mesh insertion compared to partially absorbable mesh insertion for anterior vaginal wall prolapse repair

**DOI:** 10.1590/S1677-5538.IBJU.2019.0141

**Published:** 2019-12-17

**Authors:** Elad Leron, Mona Toukan, Polina Schwarzman, Salvatore Andrea Mastrolia, Jacob Bornstein

**Affiliations:** 1 Department of Obstetrics and Gynecology, Soroka University Medical Center, Ben Gurion University of the Negev, Beer Sheva, Israel; 2 Department of Obstetrics and Gynecology, Galilee University Medical Center, Bar Ilan University, Nahariya, Israel;; 3 Department of Obstetrics and Gynecology, Ospedale dei Bambini “Vittore Buzzi”, University of Milano, Milano, Italy

**Keywords:** Pelvic Floor, Postoperative Complications, Surgical Mesh

## Abstract

**Objective:**

To evaluate long-term (5-10 years) outcomes of Minimally Invasive Surgical (MIS) kit insertion with Prolift^®^ (non-absorbable) mesh compared to the use of Prolift M^®^ (partially absorbable), for anterior vaginal wall prolapse repair.

**Study design:**

In this retrospective study we compared women undergoing MIS kit Prolift^®^ insertion (n=90) vs. Prolift M^®^ insertion (n=79) for anterior vaginal wall prolapse repair between 2006 and 2012 at our Institution. A number of 169 women fulfilled the inclusion criteria and were included in the study.

**Results:**

During the study period 128 women (76%) completed full follow-up; of them 58 (73%) following MIS kit Prolift^®^ insertion, and 70 (88%) following MIS kit ProliftM^®^ insertion. There was no significant difference between the Prolift® and Prolift M^®^ regarding parity (3.04 vs. 2.88, p=0.506), presence of hypertension (24.1% vs. 39.1%, p=0.088), diabetes mellitus (3.4% vs. 11.6%, p=0.109), or urinary stress incontinence (39.7% vs. 47.1%, p=0.475). All participants had been diagnosed with POP grade 3 or 4 before the procedure. No significant complications during the procedure or postoperative period were identified in the study groups. The follow-up period was at least five years in duration for both groups. Both groups were comparable according to questionnaires focused on function and satisfaction.

**Conclusion:**

Patients undergoing MIS kit Prolift^®^ and Prolift M^®^ insertion for anterior vaginal wall prolapse repair had comparable early and late postoperative outcomes. No differences in patient's function and satisfaction between the two groups were identified. According to our findings, there is no superiority to either of the two studied mesh devices.

## INTRODUCTION

Pelvic organ prolapse (POP) is a common and challenging medical condition worldwide. This is an expression of a herniation process in the pelvic floor. The background for this process is supposed to be the weakness of supporting tissues. There is a continuous effort for improving the surgical methods employed for its correction, given the significance associated to high recurrences and reoperation rates. The rise and partial fall of transvaginal kits for POP repair is a challenging debate among pelvic surgeons.

Surgery for POP repair is the option for women with advanced prolapse, suffering from symptoms that significantly affect their quality of life, and for women who desire definitive treatment.

The surgical approach is intended to correct the weakness and the thinning of the supportive tissues of pelvic organs. Although the correction of POP using the natural tissues (anterior and posterior colporrhaphy) has been a common procedure until recently, it has been associated to high recurrence rates. Several studies report that patients who undergo natural tissue repair surgeries will need repeated surgery due to recurrence in 6-29% of the cases (1, 2). Therefore, the use of those defective tissues for pelvic floor support might lead to the recurrence of the prolapse (3).

At the beginning of the last century, general surgeons began to use synthetic meshes to minimize the rate of hernia relapses. Initially they used silver wires, and then switched to nylon and polypropylene meshes. Absorbable products such as Polyglactin^®^ have recently been put to use in meshes (4).

Encouraged by the successful use of synthetic meshes in general surgery, gynecologists have considered the use of meshes to correct the defects in the pelvic floor, replacing the defective connective tissue considered the causal factor of this pathologic process.

At first, only non-absorbable meshes were used for POP repair. Later, partially absorbable meshes were introduced. Unsuccessful attempts to use completely absorbable implants proved that this was not a viable treatment. Today, non-absorbable and partially absorbable meshes are used.

Synthetic meshes are classified according to the size of the pores, the number of strands in the thread, the type of thread, the shape of the threading and the ratio of the weight to the area of the implanted mesh. Meshes with low weight /surface ratio, and large, single-wire perforations with elasticity of 20-35% that match the elasticity of the surrounding tissue have greater chances for success (5). Reported complications in association with meshes include pain, infection, and the penetration of the vaginal, intestinal, or urinary tract walls. Eventual mesh erosions and shrinkage may provoke an extensive fibrosis and lead to pelvic pain, dyspareunia and dyschezia (6).

The acute and chronic response of the tissues against the inserted foreign body is a major cause of those complications. The smaller mass of the mesh in the vagina will decrease the intensity of the inflammatory process, and will reduce the frequency and intensity of complications (6, 7).

The final weight of the partially impregnated mesh (Prolift M^®^) is 31g/m^2^, three months after implantation, compared to 45g/m^2^ that is the weight of the non-absorbable mesh (Prolift^®^) (8).

There is also a difference in the size of the pores: 2.5mm in a non-absorbable mesh compared to 3.5mm in the partially absorbable product. Large pores allow better tissue growth into the mesh and prevent the development of infections that are not accessible to macrophages (6, 7). Different minimally invasive mesh insertion techniques were developed and reported to have successful results (9, 10). Moreover, there is data supporting the safety and efficacy of the original mesh placement technique (9, 10).

The use of partially absorbed implants has been shown to be effective and safe for the rehabilitation of the posterior section of the pelvic floor as compared to a non-absorbable product (8).

The fact that these implants are no longer manufactured and marketed contributes to the objectivity of the research, since there is no concern that the collection and analysis of the information will be affected by any bias of interest. Moreover, when there is a debate about the role of mesh implants in urogynecology the comparison of those two kits gives us valuable information about the outcome after POP repair with mesh, especially when applied by experienced pelvic surgeon.

In our study, we aimed to evaluate the long-term outcomes regarding success and complication rates following MIS kitProlift^®^ (non-absorbable) insertion compared to MIS kitProlift M^®^ (partially absorbable) insertion for anterior vaginal wall prolapse repair.

## MATERIALS AND METHODS

In this comparative retrospective study we included women with POP grade 3, according the POP Q scoring system (11, 12), who had undergone Prolift^®^ mesh insertion or Prolift M^®^ mesh insertion for vaginal anterior wall prolapse repair between 2006 and 2012.

The study was approved by institutional review board. All procedures were performed by the same experienced surgeon (MN). Women who had severe preoperative pelvic pain and those with collagen disorders were excluded. Outcomes assessed included demographic characteristics, surgical reports, early and late postoperative complications, and where obtained by using a computerized database. In addition, telephone interviews with questionnaires to evaluate patient satisfaction were conducted during 2015-2016. Women were asked about post-operative pelvic or vaginal pain, dysfunction of the lower urinary system and defecation, leakage or difficulty in passing urine or feces, and sexual function (19 questions relating to this subject). Women who reported continuous pain were asked to characterize the exact location of the discomfort, its intensity (according to a Visual Analog Scale-VAS curve) and the presence of dyspareunia (13, 14). All data concerning demographic characteristics, surgical reports and hospitalizations details were also collected. Coding was performed after assessing medical records as well as routine hospital documents.

During the follow-up analysis we evaluated the immediate, early and late complications and outcomes. The following conditions were evaluated: immediate complications were intraoperative blood transfusions and injuries of the urethra, urinary bladder, bowel, blood vessels; early complications were blood transfusions, intravenous antibiotic treatments during hospitalization; readmissions, early vaginal erosions, mesh exposure, urinary tract infection, pelvic hematoma, voiding difficulties and reoperations; late postoperative outcome were stress incontinence, leakage of urine, urinary retention, vaginal erosions, mesh exposure, maximal daily activity performance, emotional and mental status impairment, frustration, vaginal or pelvic pain (according to VAS), fecal incontinence, fecal urgency and sexual function.

The institutional review board approved the study that has been performed in accordance with the ethical standards laid down in the 1964 Declaration of Helsinki and its later amendments (approval number 0087/14/NHR, date of approval December 20th, 2014).

### Statistical analysis

To construct a sexual function index from the 19 questions relating to this subject, internal reliability was tested by Alpha Cronbach (alpha=0.955). For both study groups, demographic characteristics and sexual function score were compared, using T-test independently, and compared with a Wilcoxon sum rank test. Data from questionnaires regarding pelvic or vaginal pain, dysfunction of the urinary system and defecation, leakage or difficulty in passing urine or feces, and sexual function were compared using the Chi-square test or Fisher exact test.

## RESULTS

During the study period 169 women met the inclusion criteria; 90 women (53.3%) underwent MIS kit Prolift^®^ insertion and 79 (46.7%) underwent MIS kit Prolift M^®^ insertion. A number of 128 patients (76%) answered the questionnaires; 58 (73%) following MIS kit Prolift^®^ and 70 (88%) following MIS kit Prolift M^®^ insertion. Table-1 compares the demographic characteristics and preoperative health status of the study groups. There was no significant difference between the Prolift^®^ group and Prolift M^®^ group regarding the parity (3.04 vs. 2.88, p=0.506), presence of hypertension (24.1% vs. 1 %, p=0.088), diabetes mellitus (3.4% vs. 11.6%, p=0.109), or urinary stress incontinence (39.7% vs. 47.1%, p=0.475). In the Prolift^®^ group the mean age was 61.17±1.23 years. 65.04±0.91 years in the Prolift M^®^ group (p=0.011). All participants were diagnosed with POP grade 3 or 4 (POP-Q classification). No complications during the procedure and during postoperative period were identified in the study groups. The mean length of follow-up for the Prolift^®^ group was 9.1±0.59 years, and it was 4.9±0.59 years for Prolift M^®^ group.

**Table 1:** Demographic, clinical preoperative, and perioperative characteristics; assessment of women's function and satisfaction.

Demographic and clinical preoperative characteristics			
Characteristic	Prolift M^®^ group	Prolift^®^ group	P value
	(n=79)	(n=90)	
Age	65.04±0.91	61.17±1.23	0.011
Parity	2.88±1.089	3.04±1.084	0.506
Hypertension	27 (39.1)	14 (24.1)	0.088
Diabetes Mellitus	8 (11.6)	2 (3.4)	0.109
Urinary Stress Incontinence	33 (47.1)	23 (39.7)	0.475
**Perioperative characteristics of the study groups**			
**Characteristic**	**Prolift M^®^ group**	**Prolift^®^ group**	**P value**
	**(n=79)**	**(n=90)**	
POP Grade 3-4 before procedure	79 (100)	90 (100)	0.533
Complications during procedure	0 (0)	0 (0)	n/a
Mean length of follow-up (years)	4.9±0.87	9.1±0.59	<0.001
**Mean length of follow-up (years)**			6.8±2.2
**Women's function and satisfaction according to telephone interviews**			
**Characteristic**	**Prolift M^®^ group**	**Prolift^®^ group**	**P value**
	**(n=70)**	**(n=58)**	
Urgency (moderate/severe)	13 (21.4)	15 (22.4)	1.000
Urge incontinence (moderate/severe)	12 (17.1)	6 (10.3)	0.315
Stress incontinence (moderate/severe)	7 (10)	3 (5.2)	0.347
Leakage of urine (moderate/severe)	9 (12.9)	2 (3.4)	0.110
Urinary retention (moderate/severe)	3 (4.3)	3 (5.2)	1.000
Maximal Daily activity	56 (80)	49 (86)	0.481
Emotional and mental status impairment (moderate/severe)	3 (4.3)	2 (3.4)	1.000
Frustration (moderate/severe)	2 (2.9)	2 (3.4)	1.000
Vaginal/pelvic pain	0 (0)	1 (1.7)	1.00
Fecal incontinence	8 (11.4)	5 (8.6)	0.77
Fecal urgency	0 (0)	1 (1.7)	0.703
Fecal urgency with incontinence	1 (1.4)	4 (6.9)	0.175
Sexual function	2.350±0.82	2.395±0.79	0.809

n/a, not available

Data is presented as mean±standard deviation, number (percentage)

The mean length of follow-up in both groups was 6.8±2.2 years. While there is a difference in the length of follow-up, and for assessing the objective to evaluate the complications and patient satisfaction, the period of 5 years is long enough for those parameters, in our opinion. Both groups were comparable according the questionnaires focused on function and satisfaction (Table-1).

## DISCUSSION

We have found that women who had undergone MIS kit Prolift^®^ and MIS kit Prolift M^®^ insertion for anterior vaginal wall prolapse repair experienced comparable early and late postoperative identified. Although mesh implants were found to be associated with erosions and shrinkage consequently followed by postoperative pain in the pelvis or vaginal area, or dyspareunia (3) other studies report that mesh insertion is a safe and effective procedure with a high cure rate. There is a consensus regarding the role for synthetic mesh implants in women with recurrent prolapse (15). Favorable subjective and objective outcomes were found for long-term outcomes after transvaginal mesh insertion for POP (16). A long-term cohort study of surgery for recurrent prolapse, reported that a vaginal mesh repair using a non-absorbable trans-obturator mesh has improved satisfaction compared to an anterior colporrhaphy (17).

Women offered with MIS kit insertion have to be carefully selected and the procedures have to be performed by expert surgeons (18) since complications may arise from poor surgical training and inadequate surgical experience. Using the Prolift^®^ Mesh, the mass of the mesh and the local inflammatory process in the graft area should also be taken into account, as they might contribute to complications (19). To reduce the weight of the implant, Prolift M^®^ mesh was developed. A significant portion of its mass is absorbed after 90 days of surgery and this innovation was aimed to reduce the post-procedural inflammatory process and its following complications.

We found that there was no difference regarding complication rate and patient satisfaction in the group treated with Prolift^®^ compared to women treated with Prolift M^®^ mesh. During our study we did not have major complications in the study groups. The minimal complications rate in our study population was very low.

In addition, no difference regarding complication rate and patient satisfaction between the groups treated with Prolift^®^ and Prolift M^®^ was found.

Continuous search of better materials for mesh implants is in progress. Studies, which demonstrate the improved tissue integration and angiogenesis with an elastic, estradiol releasing polyurethane material designed for use in pelvic floor repair have been recently published (20).

The main strength of our study is that all procedures were performed by an experienced single surgeon. Also, it may be a reason for low complication rate, in our opinion. In addition, the follow-up period was significantly long.

Nonetheless, several limitations should be addressed when considering our results. This study is restricted by its retrospective design. There was low compliance among the patients asked to participate in the questionnaires.

Although Prolift^®^ and Prolift M^®^ implants are no longer used, the possible insights from our study results may shed light on the benefits of other semi-absorbed light-weight implants currently in use.

## CONCLUSIONS

Carefully selected women can benefit from synthetic mesh implants with minimal complication rate, as was shown for Prolift^®^ and Prolift M^®^ in our study and others recent studies regarding transvaginal meshes. Therefore, performing transvaginal mesh repair with the appropriate technique should be considered for selected cases. As long as the manufacturing of a new kind of mesh continues, more clinical studies are needed for evaluation of the efficacy and safety of those implants.
